# Highlights from the 36th European Society of Surgical Oncology Congress (ESSO 36), 14–16 September 2016, Kracow, Poland: optimising European cancer surgery

**DOI:** 10.3332/ecancer.2017.728

**Published:** 2017-03-23

**Authors:** Danuta Lichosik

**Affiliations:** IEOEDUCATION, School of Robotic Surgery, European Institute of Oncology, Milan, Italy

**Keywords:** cancer, surgical oncology, new technology, mini invasive surgery, robotic surgery, research, education

## Abstract

The ESSO Congress is the event for all surgeons with an interest in surgical oncology. The congress is a gathering for the surgical oncology community from all around the world to meet and gain an insight into state-of-the-art technology, latest healthcare services, and solutions within their field.

The ESSO 36 congress gathered over 750 participants from 58 countries. With over 100 speakers, the scientific programme featured 18 scientific symposia including a joint symposium with the American Society of Surgical Oncology (SSO), 6 multidisciplinary sessions including joint sessions with CIRSE (Cardiovascular and Interventional Radiological Society of Europe) and JCOG (Japanese Clinical Oncology Group), 8 meet-the-expert sessions, 5 debates, 13 proffered paper sessions, and 2 video sessions.

The 36th ESSO Congress (14–16 September 2016, Krakow, Poland) was held in partnership with the Polish Society of Surgical Oncology and welcomed delegates from 58 countries. During the congress a wide range of topics were discussed and there were exceptional speakers from Europe and beyond. The congress focused on ‘Optimising European Cancer Surgery’ with many sessions and discussions covering issues related to quality assurance, guidelines, and strategies to bridge the gap between minimum standards of care across Europe. Among the various sessions of interest, the symposium ‘Surgical Oncology in Central-Eastern Europe: Problems and Solutions’ chaired by Peter Naredi, ECCO President, and the Congress Co-Chair Piotr Rutkowski was widely attended and further became the congress’ *fil rouge*. The specific issues of Central and Eastern Europe regarding for e.g. limited healthcare resources, the recognition of surgical oncology as a separate medical specialty, and possible solutions for a unified oncological surgery in Europe were discussed during this symposium. Experts from Russia, Latvia, Hungary, and Poland also joined in.

## Introduction

The congress co-chairs Riccardo Audisio (UK), Piotr Rutkowski (Poland), and Krzysztof Herman (Poland) opened the congress by welcoming all participants and stressing together with the ECCO President, Peter Naredi, the importance of multidisciplinarity in improving the cancer patient’s journey. All the topics discussed over the three days highlighted the significance of a truly multidisciplinary approach in cancer management. This approach is based on the belief that every cancer patient deserves a personalised therapeutic plan, where all involved specialists can contribute their knowledge and surgery can be combined with other treatments for an optimal outcome.

The latest developments and key issues in surgical oncology was discussed during many of interactive sessions to highlight the significance of a multidisciplinary approach for management of cancer patient care.

## Programme highlights

To optimise the delegates’ congress experience, based upon direct feedback from the previous congress, the following popular sessions were included:

- Debates.
- Short clinical case studies on specific topics were presented and the audience had a chance to vote before and after the debate.
- Expert video sessions.
- Experts presented videos featuring surgical technique and procedures highlighting best practices or latest developments.
- Meet the Expert Sessions–these sessions were specially formulated to provide delegates with an opportunity to learn from key experts in an intimate setting to promote interaction and discussion. The sessions were open to all attendees but seats were limited.
- Multidisciplinary sessions.
- Novel sessions with an emphasis on the bench to bedside aspect encompassing various disciplines to discuss key clinical science issues.

### Keynote address

Prof Santiago Gonzalez-Moreno, ESSO President, Medical Director and Head of Surgical Oncology, MD Anderson Cancer Centre Madrid, Spain, during the opening ceremony of Congress said ‘It is a pleasure and a great honour for me to become the new President of the European Society of Surgical Oncology. Some years ago, as a regular ESSO member, I would never have expected to take on this important role in our society, but I am here today to serve our members so that we can all deliver the best surgical oncology care for our patients’.

First of all, he sincerely thanked Prof Riccardo A Audisio (UK) who had skillfully led ESSO during the past two years and congratulated the new President-elect, Dr Tibor Kovacs (UK-Hungary), the new Treasurer Prof Domenico M D’Ugo (Italy), and also their renowned colleagues who also joined the ESSO Board of Directors for the first time on this occasion: Prof William Allum (UK), Dr Hassan Malik (UK), Prof Michel Rivoire (France), and Dr Isabel T. Rubio (Spain).

Reliability, solidity, and dedication are the major strengths of ESSO which is proven by the amount of interesting opportunities already offered to the ESSO community in the fields of education, research, and leadership in the multidisciplinary treatment of cancer. The years ahead will be crucial for ESSO to position itself as a key player in the changing environment of cancer care in Europe, to underline the crucial role of specialised surgeons in the treatment of cancer, and to ensure the economic sustainability of the organisation. ESSO will carry on consolidating the trust of their members in what ESSO can do to support them in their daily clinical and research work by showing a greater sensitivity to their needs and by encouraging a frequent and bidirectional communication with them.

## Proffered papers

The evolution of surgical oncology techniques for a better quality of life for patients was the focus of the ‘Endpoints in Surgical Oncology’ session chaired by S Bonvalot (France) and W Ceelen (Belgium) on the first day of the congress. Several experts discussed the importance of the co-ordination of surgery with other treatments, the role of surgery in metastatic diseases, the validated use of laparoscopy /robotic surgery and reconstructive surgery, and many examples were presented.

The multidisciplinary session ‘Translating Basic Science in Cancer Surgery to Clinical Care’ demonstrated the central role of surgeons in giving cancer patients the optimal care and chances for a good quality of life (QoL)after treatment. Surgical oncologists are nowadays in charge of assessing the risks involved with each treatment option on the basis of recent translational research knowledge. With the contribution of K Soreide (Norway) and P Naredi (Sweden), participants in this session, the potential of using biomarkers, the importance of certain tumour mutations and the cancer immunology in deciding which surgical procedure to use, the extent of resection margins, and other aspects of the daily work of a surgical oncologist were brought forth. Their views were exchanged with all.

At the multidisciplinary session ‘Robotic Surgery in Gastrointestinal Cancer: Hype or Hope’ , participants had the opportunity not only to evaluate the potential benefits but also the controversial aspects of robotics in surgical oncology. The future directions of new robotic technologies was the core of the discussion which benefitted from the participation of expert robotic surgeons including D Perez (Germany) and D D’Ugo (Italy).

Chaired by S Evrard (France) and AE Giuliano (USA), the scientific symposium on clinical research in surgical oncology debated the overlooked role of surgery in cancer research. He pointed out to the cover story recently by the Cancer World ‘The invisible cure. Should we be talking more about cancer surgery?’. The presentation also involved how surgeons publish very few randomised controlled trials (RCTs) and only 5% of funding in oncology goes to surgical trials.

During the multidisciplinary session with the Cardiovascular and Interventional Radiological Society of Europe (CIRSE), the lecture on the developments from evidence-based interventional oncology from ESSO Past President G Poston (UK), also raised considerable attention.

The inspirational lectures from a few key ESSO speakers on how surgical oncology will evolve in the next 15 years and on the role of cancer surgeons as the gatekeeper of good cancer care kicked off an interesting discussion during the scientific symposium ‘The Future Environment for Cancer Surgeons’.

The ‘Meet the Expert’ sessions on different topics from oncoplastic breast techniques to the controversial Hyperthermic Intraperitoneal Chemotherapy (HIPEC), from perihilar cholangiocarcinoma to extralevator abdominoperineal excision or abdominosacral amputation of the rectum along with the ‘Expert Video’ sessions on cancer of the oesophago-gastric junction and breast cancer contributed to the success of the congress.

During dedicated plenary sessions, the prestigious ESSO awards were presented to illustrious experts in recognition of their outstanding contribution to surgical oncology on this occasion:

- Luigi Cataliotti (Italy) was awarded with the ESSO Lifetime Achievement Award;
- Andrzej Kułakowski (Poland) was awarded with the ESSO-PSSO Award;
- Niall O’Higgins (Ireland) was awarded with the ESSO Medal in recognition of his continuous efforts towards the improvement of the education and training of young surgeons and of his investment in ESSO’s successful growth and internationalisation;
- The EJSO Award Lecture 2016 awarded to Daniel G Coit (USA), President of the American Society of Surgical Oncology (SSO), for his work on lymph nodes.

Several young surgeons were rewarded for their presentations, posters, initiatives, and videos during the congress. The ‘Niall O’Higgins Award for the best proffered paper’ went to YW Kim (Korea) for his presentation on ‘Laparoscopy-assisted versus open D2 distal gastrectomy for advanced gastric cancer’.

## Posters

The ‘ESSO Awards for the Best Posters’ were presented to:

- 1st prize to Karol Polom (Italy) for the poster on ‘Heterogeneity of new molecular gastric cancer classifications. Clinico-pathological characteristic’;
- 2nd prize to Karl Mrak (Austria) for the poster on ‘Diverting ileostomy versus no diversion after low anterior resection for rectal cancer: A prospective, randomized, multicentre trial’;
- 3rd prize to Kristien Keymeulen (the Netherlands) for the poster on ‘The effect of preoperative breast MRI on the surgical management of ductal carcinoma in situ and the risk of contralateral breast cancer’ (all posters are available in a pdf format from the ESSO congress webpage).

Finally, the ESSO-BSSO International Exchange Programme was also showcased at ESSO36, with an award to the first visiting observer, Raquel de Fátima Quintino (Brazil).

The ‘ESSO Best Video Poster Prizes” were awarded to:

- 1st prize to B Bodanese (Brazil) for the video on ‘Body Gastric Cancer: Laparoscopic Total Gastrectomy’
- 2nd prize to A Lukashenko (Ukraine) for the video on ‘Pancreatiocoduodenectomy with partial resection of pancreatic body and tail for a multifocal neuroendocrine tumours’
- 3rd prize to A Zhygulin (Ukraine) for the video on ‘Oncoplastic lumpectomy with LICAP perforator flap reconstruction + SLNB’

## Conclusion

ESSO 36 provided a unique educational platform with a top-quality programme and presentation from exceptional speakers who contributed to the dissemination of the best knowledge and expertise to the ESSO community. Furthermore, ESSO will increasingly take into account the priorities of patient representatives via the recently launched ‘Patients’ Advisory Committee’ (PAC@ESSO) within our structure–and young professionals and via the already very active and successful ESSO Young Surgeons and Alumni Club (EYSAC). The congress also saw the launch of the new European School of Soft Tissue Sarcoma Surgery which is an important ESSO initiative in collaboration with a CTOS (Connective Tissue Oncology Society) and some European reference centres specialised in the surgical treatment of soft tissue sarcoma. It is a rare pathology where more structured knowledge is greatly needed.

Additionally, ESSO will reinforce and further promote an active collaboration, communication and exchange with other European oncology scientific societies especially ESTRO. Among all it is fair to say that ESTRO has always been a very valuable partner throughout the years.

The ESSO President pointed out during his presentation that general health and specific cancer care inequalities are a reality at the European level. There are huge differences even in regards to the minimal standards for quality surgical care provided to cancer patients across the whole continent. Experienced colleagues from the ESSO Board and from the Education and Training Committee will continue the ESSO educational portfolio of facilitating training courses, fellowships, specialised schools, and examinations throughout the continent.

Surgical oncologists will also take a more active role in the definition of a pan-European ‘oncopolicy’. This an important area where there is a need to stand up and stress the key role on how significant specific contribution of good quality cancer surgery is to the diagnosis and management of cancer patients. ESSO’s participation in the recent ECCO project involving the essential requirements for quality cancer care is the first important step in this direction.

Concluding his first message as President, Prof Santiago Gonzalez-Moreno invited all surgical oncology specialists to ESSO37 (27 January 2017, Amsterdam, The Netherlands) which is a one-day conference focusing on breast and colorectal cancer surgical oncology.

## Figures and Tables

**Figure 1.**
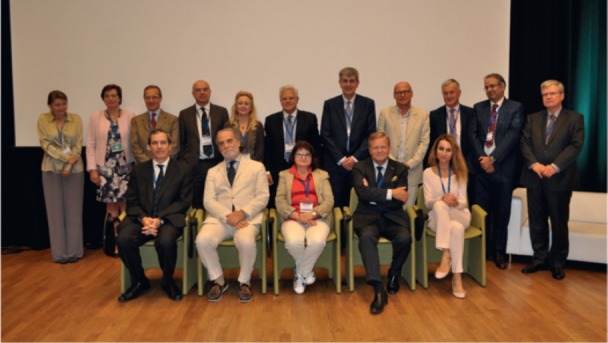
A picture of the ESSO Board of Directors taken in Krakow on the occasion of ESSO 36, September 2016.

**Figure 2.**
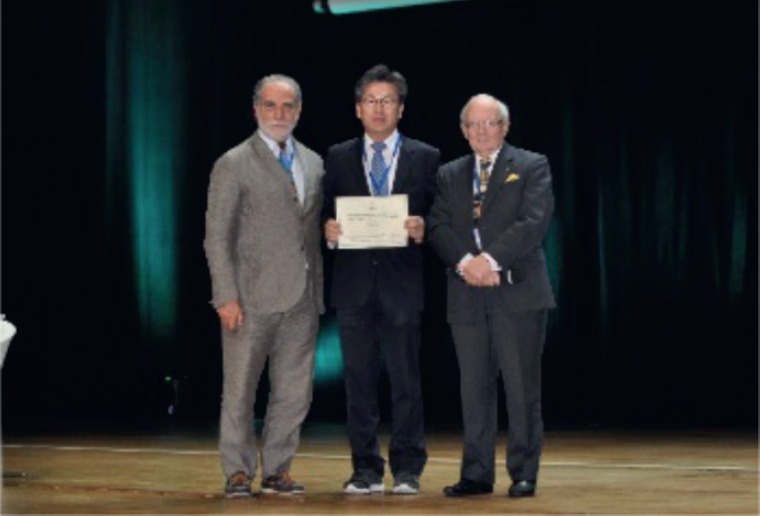
Prof Niall O’Higgins and Prof Riccardo A Audisio giving YW Kim the “Niall O’Higgins Award for the best proffered paper”.
